# High glucose and palmitic acid induces neuronal senescence by NRSF/REST elevation and the subsequent mTOR-related autophagy suppression

**DOI:** 10.1186/s13041-022-00947-2

**Published:** 2022-07-18

**Authors:** Wen-Jiao Xue, Cheng-Feng He, Ren-Yuan Zhou, Xiao-Die Xu, Lv-Xuan Xiang, Jian-Tao Wang, Xin-Ru Wang, Hou-Guang Zhou, Jing-Chun Guo

**Affiliations:** 1 Department of Translational Neuroscience, Jing’an District Centre Hospital of Shanghai, State Key Laboratory of Medical Neurobiology and MOE Frontiers Center for Brain Science, Institutes of Brain Science, Fudan University, Shanghai, China; 2 Department of Geriatric Neurology of Huashan Hospital, National Clinical Research Center for Aging and Medicine, Fudan University, Shanghai, China; 3 Department of Urology, Jing’an District Central Hospital, Fudan University, Shanghai, China

## Abstract

Cell senescence is a basic aging mechanism. Previous studies have found that the cellular senescence in adipose tissue and other tissues, such as the pancreas, muscle and liver, is associated with the pathogenesis and progression of type 2 diabetes; however, strong evidence of whether diabetes directly causes neuronal senescence in the brain is still lacking. In this study, we constructed a high glucose and palmitic acid (HGP) environment on PC12 neuronal cells and primary mouse cortical neurons to simulate diabetes. Our results showed that after HGP exposure, neurons exhibited obvious senescence-like phenotypes, including increased NRSF/REST level, mTOR activation and cell autophagy suppression. Downregulation of NRSF/REST could remarkably alleviate p16, p21 and γH2A.X upregulations induced by HGP treatment, and enhance mTOR-autophagy of neurons. Our results suggested that the diabetic condition could directly induce neuronal senescence, which is mediated by the upregulation of NRSF/REST and subsequent reduction of mTOR-autophagy.

**Supplementary Information:**

The online version contains supplementary material available at 10.1186/s13041-022-00947-2.

## Introduction

Diabetes is a metabolic disease characterized by chronic hyperglycemia. The prevalence of diabetes increases with aging [1]. Patients with diabetes are more likely to develop age-related complications, such as cognitive impairment, Alzheimer's disease, cardiovascular disease, visual impairment and renal dysfunction [2, 3]. Young patients with diabetes show similar multiple organ system dysfunction to normal aging, suggesting that diabetic patients may have experienced a pro-ageing state [4].

Cell senescence is one of the causative processes of aging, which can be caused by telomere erosion, DNA damage, oxidative stress and oncogene activation [5]. Traditionally, cellular senescence is described as the state of cell cycle arrest. Recently, the phenotypes related to cellular senescence have extended beyond growth arrest, including changes in cell metabolism, cytokines secretion, epigenetic regulation and protein expression [6]. Senescent cells are usually characterized by the enhancement of oxidative stress and senescence-associated β-galactosidase (SA-β-gal) activity, the up-regulation of p21 and p16, and the accumulation of DNA damage [7].Though neurons are not subject to replicative senescence, accumulating evidences have reported that a series of senescence-like phenotypes do exist in neurons, such as the SA-β-gal activation, telomere shortening and DNA damage responses [8]. However, the mechanism underlying the senescence-like changes in neurons is still unclear.

High circulating glucose, altered lipid metabolism, and growth hormone axis perturbations can promote the senescent cell formation [9]. Previous studies have demonstrated that insulin resistance directly increases the proportion of senescent β cells, and that hyperglycemia causes vascular aging [10, 11]; however, whether hyperglycemia promotes neuronal senescence remains unclear. A recent study has demonstrated that the differentially expressed genes of cortical neurons in patients with type 2 diabetes mellitus are enriched in the signal pathway of cell senescence [12], which suggests that the brain neurons may also appear senescence-like phenotypes under the neuropathological process of diabetes. Therefore, in the present study, we wondered whether diabetes directly causes the cell senescence in neurons.

In our previous studies, we have found that diabetes increases the protein level of neuron restrictive silencer factor (NRSF), also known as repressor element-1-silencing transcription factor (REST), which then participates in the neuronal injury under hyperglycemia [13]. NRSF/REST is a member of the zinc finger protein transcription factor family and plays complex regulatory roles in the physiological and pathological processes of the nervous system [14]. Interestingly, it has been reported that elevated nuclear levels of NRSF/REST is a characteristic of neuronal senescence in healthy aging processes [15]. Therefore, we hypothesize that the diabetes-induced elevation of NRSF/REST contributes to the neuronal aging.

In the present study, we used high glucose and palmitic acid (HGP) to simulate the diabetic environment in PC12 neuronal cells and primary mouse cortical neurons (PCNs). Our results showed that HGP treatment induces evident senescence-like phenotypes in neurons, accompanied by NRSF/REST upregulation and neuronal autophagy disturbances. Meanwhile, down-regulation of NRSF/REST remarkably promotes cellular autophagy and alleviates the senescence-like phenotypes induced by HGP, indicating that elevation of NRSF contributes to diabetes-related pro-aging in neurons.

## Material and methods

### Chemicals and antibodies

MHY1485 (MCE, HY-B0795), Bafilomycin A1 (BafA1, MCE, HY-100558), anti-β-actin (Proteintech, 20536-1-AP), anti-p16 (SantaCruz, sc-1661), anti-p21 (SantaCruz, sc-6246), anti-γH2A.X (CST, 2577), anti-MAP2 (Abcam, ab183830), anti-MAP2 (Abcam, ab11268), mTOR (CST, 2983), p-mTOR (CST, 5536), LC3B (Affinity, AF4650), anti-p62 (CST, 23214), anti-ATG7 (Proteintech, 67341-1-lg), anti-REST (Novus, NBP1-82399).

### Cell culture and treatments

Highly differentiated PC12 cells (Shanghai Fuheng Biology) were cultured in DMEM/F12 medium (Tianjin Hao Yang Biological Manufacture) containing FBS (Thermo Fischer Scientific) and streptomycin/penicillin (Thermo Fischer Scientific) at 37 °C and 5% CO_2_. CCK-8 assay kit (Beyotime Biotechnology) was used for cell activity detection and finally treated PC12 cells with 100 mmol glucose and 200 µmol palmitic acid for 24 h (Additional file 2: Fig. S2).

Primary cortical neurons were obtained from embryonic day 16–18 C57BL/6 mice (Shanghai Jiesijie Experimental Animal). The cortical tissues were isolated on ice and digested with trypsin (Thermo Fischer Scientific) at 37℃for 20 min. After tissue blowing, the obtained cell suspension was inoculated on the cell culture plate or slide coated by poly-l-lysine (Sangon biotech). The cells were cultured at 37 °C and 5% CO_2_ with neurobasal medium (Thermo Fischer Scientific) supplemented by B27 (Thermo Fischer Scientific) and L-glutamine (Sigma). On the fourth day, the cells were cultured in the medium containing 75 mmol glucose and 100 µmol palmitic acid for 24 h, then the cells were collected for further experiments.

### Knockdown of NRSF/REST

NRSF/REST level was knocked down by transfection of AAV shRNA expressing vectors containing NRSF/REST shRNA according to our previously described methods [13]*.*

### Western blot analysis

The cell lysate was added with loading buffer (Epizyme Biotech) solution and boiled at 100 ℃ for 10 min. The prepared samples were separated by SDS–polyacrylamide gel electrophoresis, and then transferred to a polyvinylidene fluoride membrane (Millipore) after protein separation. The membrane was blocked with blocking solution at room temperature for 1 h, and then incubated with primary antibodies at 4 ℃ overnight. After PBST was used to wash membrane for three times, HRP labeled secondary antibody (CST) was added and incubated at room temperature for 1 h. PBST was also used to wash membrane and chemiluminescence detection kit (Epizyme Biotech) was used for visualization of protein bands. The protein bands were quantitatively analyzed by Image J software.

### Immunofluorescence staining

After the cells were washed with PBS, 4% PFA was added to fix the cells at room temperature for 20 min. After fixation, the cells were washed with PBS for 3 times. 0.3% PBST was added to permeate cells for 20 min on ice. PBS was used to wash cells for 3 times. After that, the cells were blocked at room temperature for 1 h. Primary antibody was added at 4 ℃ overnight. Then PBS was used to wash cells for 3 times and secondary antibody conjugated with Alexa488 or 594 (Jackson) was added for 2 h avoid light at room temperature. After PBS washing for 3 times, slide was sealed with tablet containing DAPI and photographed by fluorescence microscopy.

### Assay of SA-β-gal activity

SA-β-gal activity was detected by senescence β-galactosidase staining kit (Beyotime Biotechnology) according to the manufacturer's instructions. After incubation at 37 ℃ overnight, the percentage of positive cells was recorded under light microscope.

### Statistical analysis

All data were represented as the mean ± SD. Results were analyzed by using GraphPad Prism 9.0 software. Unpaired t-test was used for the comparison between the two groups, one-way ANOVA and the two-way ANOVA was used for multiple comparisons. p < 0.05 was considered statistically significant.

## Results

### HGP induces senescence-like phenotypes in neurons

Firstly, we performed CCK-8 detection to investigate the effect of HGP concentration on neuronal viability according to previous studies [13]. As showed in Additional file 1: Fig. S1a and S1b, we found that after 24 h exposure with 75 mmol glucose/100 µmol palmitic acid (G75P100) on PCNs or 100 mmol glucose/200 µmol palmitic acid (G100P200) on PC12 cells, the neuronal viability was significantly reduced when compared with that in control group.

**Fig. 1:**
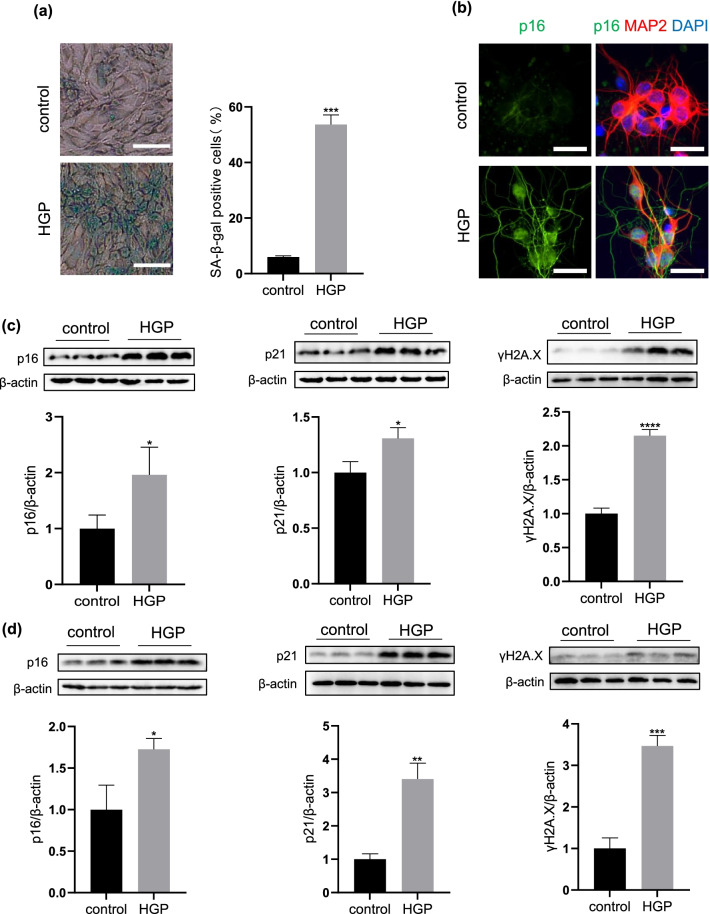
HGP induce neurons to exhibit senescence-like phenotypes **a** Increase of SA-β-gal activity in PC12 cells induced by HGP. Scale bar, 50 μm. **b** Immunofluorescence staining of p16 in PCNs at control and HGP. Scale bar, 25 μm. **c** The expression of senescence marker proteins p16, p21 and γH2A.X in PC12 cells at control and HGP. **d** The expression of senescence marker proteins p16, p21 and γH2A.X in PCNs at control and HGP. Data are mean ± SD. n = 3 independent experiments for (**a**), (**c**) and (**d**), n = 4 independent experiments for (**b**). *p < 0.05, **p < 0.01, ***p < 0.001, ****p < 0.0001. Unpaired t test for (**a**), (**c**) and (**d**)

Next, we evaluated the senescence-like phenotypes in neurons under these appropriate HGP conditions. SA-β-gal activity has been widely used as a classical marker of cellular senescence. As showed in Fig. 1a, we found that the percentage of SA-β-gal positive cells was remarkably elevated in PC12 cells after HGP treatment (p < 0.001). DNA damage response is one of the main determinants of cell aging. The DNA damage-induced upregulation of cell cycle suppressor proteins such as p16 and p21 has been reported to participate in cellular senescence as well. By using western blotting analysis, we also found that after HGP treatment, the protein levels of p16 and p21 were significantly increased in PC12 cells (p < 0.05, Fig. 1c). Meanwhile, the DNA damage marker γH2A.X level was also remarkably increased (p < 0.0001, Fig. 1c). Interestingly, similar senescence-like phenotypes also appeared in PCNs after the HGP treatment. As showed in Fig. 1b, the p16 immunofluorescence positive particles remarkably increased after HGP exposure. Similarly, the protein levels of p16 (p < 0.05), p21 (p < 0.01) and γH2A.X (p < 0.001) were also significantly upregulated (Fig. 1d). These findings suggested that HGP exposure could directly induce typical senescence-like phenotypes in neurons.

### HGP activates the mTOR signaling and inhibits the autophagic flux in neurons

**Fig. 2:**
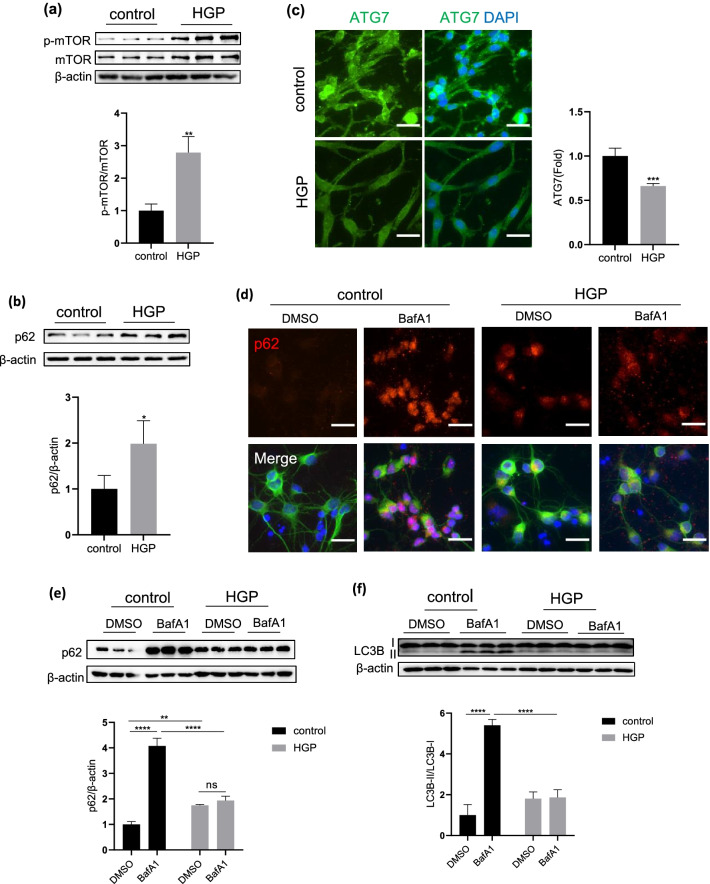
The induction of HGP activated the mTOR signal pathway and inhibited the autophagy function (**a**), (**b**) Western blotting image of p-mTOR, mTOR and p62 in PC12 cells at control and HGP. **c** Immunofluorescence staining of ATG7 in PC12 cells at control and HGP. **d** Immunofluorescence staining of p62 in PCNs. Scale bar, 25 μm. **e**, **f** Detection of autophagy flux in PC12 cells under the treatment of BafA1(50 nmol,4 h). Data are mean ± SD. n = 3 independent experiments for (**a**), (**b**), (**d**), (**e**) and (**f**), n = 4 independent experiments for (**c**). *p < 0.05, **p < 0.01, ***p < 0.001, ****p < 0.0001. Unpaired t test for (**a**), (**b**) and (**c**), two-way ANOVA for (**e**) and (**f**)

Previous studies have demonstrated that mTOR signaling might be related to the process of cellular senescence [16], therefore, we detected the changes of mTOR phosphorylation in neurons after the induction of HGP. As showed in Fig. 2a, when compared with the control group, HGP treatment significantly increased the level of mTOR phosphorylation (p < 0.01), indicating that the mTOR signaling is activated by HGP exposure. Since the mTOR signaling participates in a series of key cellular processes, including cell metabolism and autophagy in neurodegeneration and aging [17], we then detected the changes of cellular autophagy in HGP-treated neurons. As showed in Fig. 2b, we found that the p62 level was significantly increased in HGP-treated neurons (p < 0.05), demonstrating a reduction of autophagy. We then performed immunofluorescence staining of the autophagosomes marker ATG7 and found that the expression level of ATG7 was significantly decreased in HGP neurons (p < 0.001, Fig. 2c). Furthermore, we used BafA1, an autophagy inhibitor, to detect the changes of autophagy flux in neurons. As showed in Fig. 2d, e and f, we found that under control conditions, the level of p62 and LC3B-II/LC3B-I was significantly increased after adding BafA1; whereas in HGP neurons, the p62 level and LC3B-II/LC3B-I did not change after BafA1 treatment when compared with the DMSO control neurons. These results suggested that HGP exposure could activate the mTOR signaling and inhibit the autophagy flux in neurons.

**Fig. 3:**
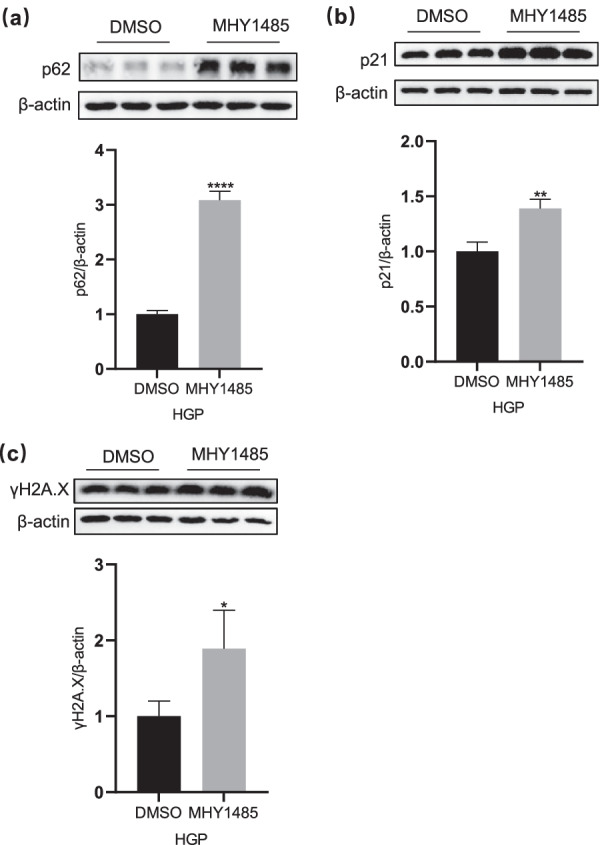
Inhibition of autophagy aggravates senescence-like phenotypes in neurons (**a**–**c**) Western blotting image of p62, p21 and γH2A.X in PC12 cells under the treatment of DMSO and MHY1485 (10 µmol, 4 h). Data are mean ± SD. n = 3 independent experiments. *p < 0.05, **p < 0.01, ***p < 0.001, ****p < 0.0001. Unpaired t test for (**a**), (**b**) and (**c**)

In order to further explore the effects of mTOR activation and autophagy inhibition on neuronal senescence-like phenotypes, we used MHY1485, an mTOR activator and autophagy inhibitor, on HGP-treated neurons. As showed in Fig. 3a, MHY1485 significantly increased the p62 level (p < 0.0001); meanwhile, the p21 and γH2A.X levels of HGP neurons were remarkably upregulated after MHY1485 treatment (p < 0.01 and p < 0.05, respectively). Therefore, these results further demonstrated that the HGP-induced mTOR activation and autophagy inhibition contributes to the neuronal senescence in HGP neurons.

### HGP upregulates and redistributes subcellular localization of NRSF/REST in neurons

**Fig. 4:**
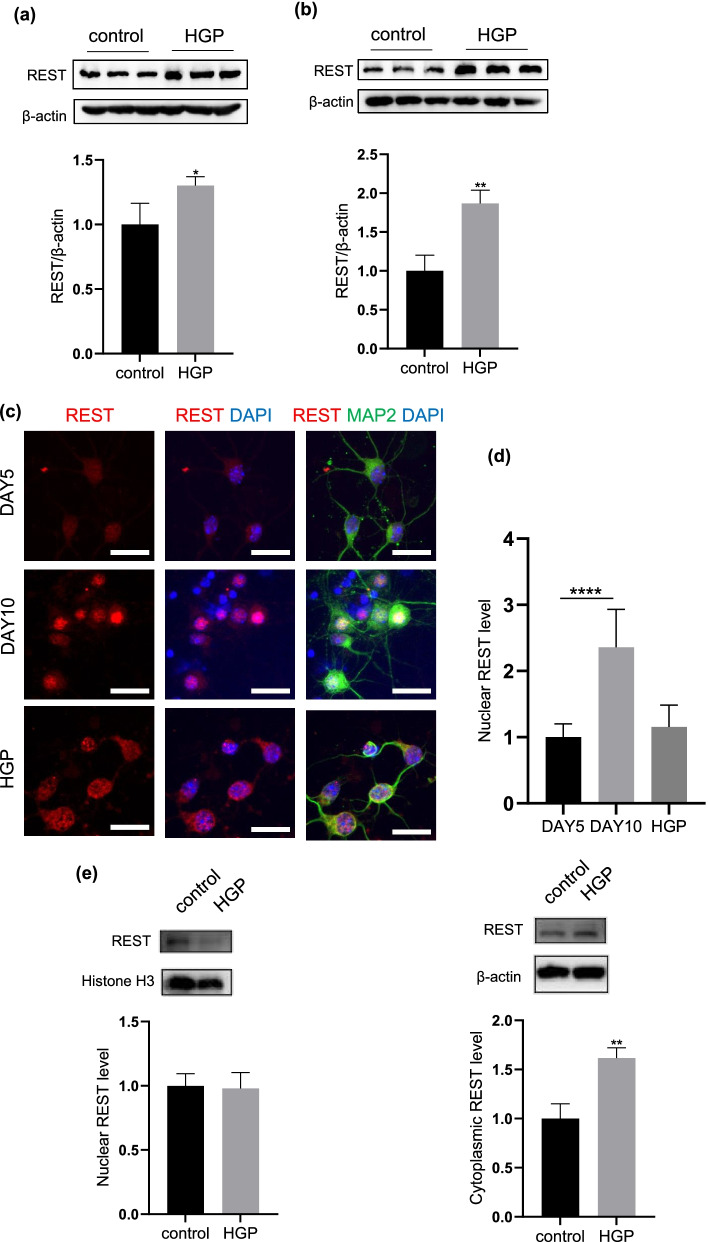
Expression and distribution of REST after treatment of HGP **a** Western blotting image of REST in PC12 cells at control and HGP. **b** Western blotting image of REST in PCNs at control and HGP. **c** Immunofluorescence staining of REST in PCNs at Day5, Day10 and HGP. Scale bar, 25 μm. **d** Nuclear fluorescence intensity of REST. **e** The expression level of REST in cytoplasm and nucleus of PC12 cells under HGP. Data are mean ± SD. n = 3 independent experiments. *p < 0.05, **p < 0.01, ***p < 0.001, ****p < 0.0001. Unpaired t test for (**a**), (**b**) and (**e**). One-way ANOVA for (**d**)

We previously found that HGP treatment could upregulate the NRSF/REST protein level in neurons [13]. In the present study, by using western blotting analysis, we also confirmed that the protein levels of NRSF/REST were significantly elevated in both the PC12 cells (p < 0.05, Fig. 4a) and PCNs (p < 0.01, Fig. 4b) after HGP exposure. To clarify the subcellular distribution changes of NRSF/REST after HGP, we performed immunofluorescence triple staining of NRSF/REST, MAP2 and DAPI on PCNs. We found that in the control neurons, the NRSF/REST positive fluorescent signals were evenly localized in both the cytoplasm and the nucleus at the Day 5 of culture and notably accumulated in the nucleus at the Day 10 of culture. However, in HGP-treated neurons, we found that the elevation of NRSF/REST positive particles existed not only in the nucleus but also in the cytoplasm (Fig. 4c, d). We detected the level of REST in the nucleus and cytoplasm of HGP-treated PC12 cells. Compared with the control group, the level of REST in the nucleus has not changed significantly, but the level of REST in the cytoplasm was significantly increased (Fig. 4e). These results indicated that unlike the normal aging process, HGP could upregulate NRSF/REST expression and redistribute its subcellular localization in neurons.

### Knockdown of NRSF/REST alleviates the HGP-induced senescence-like phenotypes

**Fig. 5:**
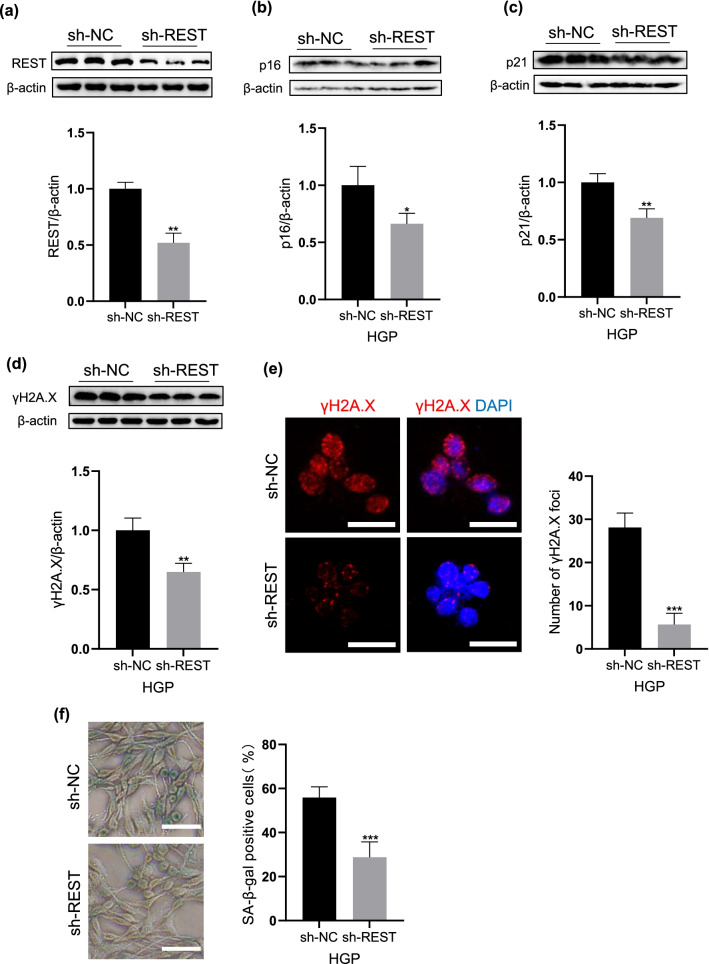
Knockdown of REST alleviates the senescence-like phenotypes **a**–**d** Western blotting image of REST, p16, p21 and γH2A.X at sh-NC and sh-REST. **e** Immunofluorescence staining of γH2A.X at sh-NC and sh-REST. Scale bar, 25 μm. **f** SA-β-gal activity at sh-NC and sh-REST. Scale bar, 50 μm. Data are mean ± SD. n = 3 independent experiments for (**a**), (**b**), (**c**), (**d**) and (**e**), n = 4 independent experiments for (**f**). *p < 0.05, **p < 0.01, ***p < 0.001, ****p < 0.0001. Unpaired t test for (**a**), (**b**), (**c**), (**d**), (**e**) and (**f**)

In order to explore the effect of NRSF/REST expression on neuronal senescence-like phenotypes, NRSF/REST level was knocked down by RNA interference. Compared with the sh-NC group transfected with empty plasmid, the expression of NRSF/REST was downregulated after sh-NRSF/REST transfection (Fig. 5a). We then detected p16 and p21 levels after HGP exposure by using western blotting analysis. As showed in Fig. 5b and c, the p16 and p21 levels were significantly decreased in sh-NRSF/REST group (p < 0.05, p < 0.01, respectively). As for the DNA damage marker γH2A.X, we found that the γH2A.X levels remarkably decreased in sh-NRSF/REST group (p < 0.05, Fig. 5d). Similarly, the γH2A.X immunofluorescence foci mainly accumulated in the nucleus and notably reduced in sh-NRSF/REST neurons (p < 0.001, Fig. 5e). Moreover, we observed that the percentage of SA-β-gal positive cells was also significantly decreased in sh-NRSF/REST neurons (p < 0.001, Fig. 5f). The above results collectively suggested that the down-regulation of NRSF/REST can alleviate the senescence-like phenotypes of HGP-treated neurons.

### Knockdown of NRSF/REST inhibits mTOR activation and promotes autophagy in HGP neurons

**Fig. 6:**
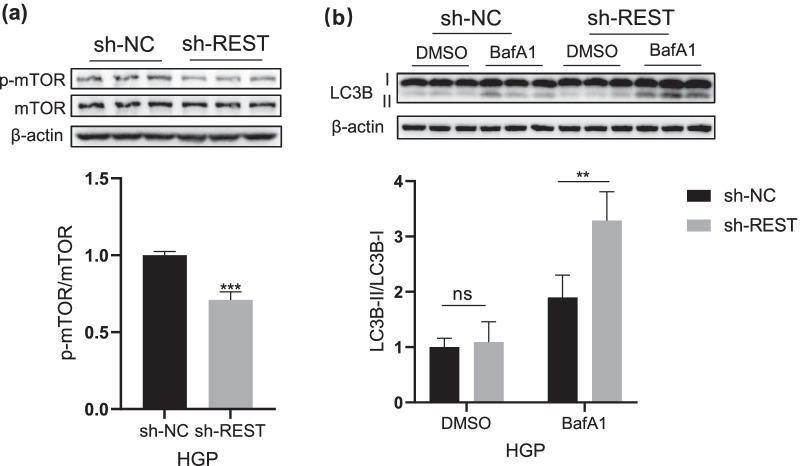
Knockdown REST inhibite the mTOR signal pathway and promoting autophagy in neurons (**a**) Western Blotting image of p-mTOR and mTOR in PC12 cells at sh-NC and sh-REST. (**b**) Detection of autophagy flux in PC12 at sh-NC and sh-REST. Data are mean ± SD. n = 3 independent experiments. *p < 0.05, **p < 0.01, ***p < 0.001, ****p < 0.0001. Unpaired t test for (**a**), two-way ANOVA for (**b**)

Previous study found that the down-regulation of NRSF/REST suppresses mTOR signaling in ovarian cancer cells [18], therefore we asked whether NRSF/REST elevation is related to mTOR activation under HGP exposure. As showed in Fig. 6a, the phosphorylation level of mTOR was significantly reduced in sh-NRSF/REST neurons. Meanwhile, we performed BafA1treatment and detected the ratio of LC3B-II/LC3B-I after the down-regulation of NRSF/REST in HGP neurons. As showed in Fig. 6b, we found that under the treatment with BafA1, the ratio of LC3B-II/LC3B-I in sh-NRSF/REST neurons was significantly higher than that in sh-NC neurons, suggesting that the decrease of NRSF/REST had a positive effect on autophagy. These results suggested that the down-regulation of NRSF/REST could alleviate the HGP-induced neuronal senescence by inhibiting the mTOR activation and promoting neuronal autophagy.

## Discussion

In the present study, we demonstrated that HGP directly induced neuronal senescence, which was mediated by the elevation of NRSF/REST and the subsequent mTOR activation and autophagy suppression. These results suggested that the role of NRSF/REST in pathological neuronal senile process might be different from that in healthy aging.

Here we showed direct evidence that the cellular senescence in the central neurons is also apparent under diabetes-like conditions. Previous studies have found that the cellular senescence in adipose tissue and other tissues, such as the pancreas, kidney and liver is associated with the pathogenesis of type 2 diabetes and further contributes to the disease [10, 19–21]; however, strong evidence of whether diabetes directly causes neuronal senescence in the brain is still lacking. Many studies have found that diabetes is accompanied by a series of changes in brain structure and function and increases risks for brain disorders such as lacunes, hippocampal atrophy and dementia, which are as similar dysfunctions as those caused by aging [22, 23]. Moreover, the anti-senile therapy could reduce neuroinflammation, restore neurogenesis and improve brain atrophy in obese animals and in Tau overexpressed old mice as well [9, 24], indicating that the diabetes-associated cognitive impairment is related to the brain senescence. Some previous studies found that brain aging is associated with substantial Ca^2+^ dyshomeostasis and that diabetes induces Ca^2+^ dysregulation in neurons [25], indicating that neuronal senescence is possibly involved in the diabetes-associated brain aging. Since cellular senescence is one of the key mechanisms of loss of proteostasis, clearance and regeneration in physiology and pathologies senility, often accompanied by changes in cell metabolism and the acquisition of senescence-specific phenotypes [7], in the present study, we evaluated several typical senescence-related markers in neurons and found that HGP could upregulate the SA-β-gal activity, enhance the p16 and p21 levels and aggravate γH2A.X formation. Cellular senescence is often triggered by irreparable DNA damage. Studies have demonstrated that serine^139^ phosphorylated H2A.X (γ-H2A.X) is a marker of DNA damage responses, which also involving several cellular processes such as p53/p21 and p16/p16INK4a-pRB activation [26]. Therefore, our results confirmed that like adipose and pancreas, the cellular senescence in the central neurons is also apparent under diabetes-like conditions. Within the present study, we have established an in vitro diabetes model in line with previous studies [13]. It is worth noting that the HGP treatment cannot fully simulate the in vivo diabetic environment and that this model needs to be further verified for relevance to the in vivo situation.

To date, mechanisms underlying neuronal senescence remain unclear. In the present study, we demonstrated that the elevation of transcriptional regulator NRSF/REST plays an important role in mediating the process of neuronal senescence under HGP treatment. These results further support our previous findings that HGP-induced upregulation of NRSF/REST contributes to the aggravated neuronal activity loss and the enhanced LDH production after HGP [13]. Previous studies found that NRSF/REST participates in the differentiation and development of neurons [27], regulates synaptic plasticity [28], maintains the self-renewal ability of neural stem cells and is also considered to be an oncogene in neurological tumor [29, 30]. Therefore, the functional abnormality of NRSF/REST is closely related to nervous system diseases [31]. Recently, studies found that in the healthy aging brain, the NRSF/REST levels increased, which mainly localized in the nucleus, inhibits the expression of apoptosis-related genes and reduces the death of neurons [32]. Moreover, NRSF/REST has been reported to reduce the nerve excitement in the normal aging process and be beneficial for prolonging the life span [33]. However, in aging-related neurodegenerative diseases, NRSF/REST may play different roles. In Huntington's disease, excessive pathological activity of NRSF/REST inhibits the expression of BDNF and results in neurodegeneration [34]. In brains with Parkinson's disease and dementia with Lewy bodies, NRSF/REST is partially sequestrated in Lewy bodies and is mostly absent from the nucleus of neurons [35]. In patients with Alzheimer’s disease, NRSF/REST appears in autophagosomes together with pathological misfolded proteins, which may be related to cognitive dysfunction in AD [32]. In the present study, our results further support the above notion that the subcellular distribution of NRSF/REST in neurons changes differently under healthy aging or HGP-related senility, that is, the increased NRSF/REST positive fluorescent particles mainly localized in the nucleus of healthy “aging” neurons after relatively long-term culture, whereas elevated in both nucleus and the cytoplasm in the HGP-treated senile neurons. The nuclear aggregation of NRSF/REST has been reported in physiological cell senescence [15]. We conjectured that the increased cytoplasm levels of NRSF/REST in HGP-induced senescence is possibly related with autophagosomes dysfunction according to the previous report [32], but the precise mechanism underlying needs further investigation.

The process of cellular senescence is accompanied by impaired proteostasis, which is also an important feature of neuropathological changes in diabetes mellitus [36]. mTOR is a serine / threonine kinase, which is the main regulator of cell metabolism. mTOR binds to a variety of associated proteins forming two different signal complexes, mTOR complex 1 (mTORC1) and mTOR complex 2 (mTORC2). mTORC1 inhibits the autophagy-initiated UNC-5-like kinase (ULK) complex by phosphorylating complex components including autophagy related gene 13 (ATG13) and ULK1/2. Autophagy induction and mTORC1 activation are closely and inversely coupled [37]. Autophagy plays an important role in the maintenance of cellular proteostasis. In aging and neurodegeneration, defective autophagy is often observed to be associated with neuronal loss and cognitive decline [38]. In our experiment, the treatment of HGP activated the mTOR signaling, resulting in dysfunction of autophagy. MHY1485 is an autophagy inhibitor that can activate mTOR and inhibit the binding of autophagosome and lysosome [39]. In our study, it was found that neurons had obvious defects in autophagy function under the treatment of MHY1485, and the cell senescence phenotype induced by HGP was further aggravated. These results suggested that the activation of mTOR and inhibition of autophagy play an important role in neurosenescence induced by HGP. We also found that unlike a recent study reporting that the NRSF/REST deficiency induces autophagy reduction and cellular senescence in neurons [40], the down-regulation of NRSF/REST decreased the level of mTOR phosphorylation and promoted autophagy in neurons under HGP treatment. These discrepancies are possibly due to the different senile status of neurons and different subcellular changes of NRSF/REST. In the present study, neuronal senescence was induced by the HGP treatment, while in Rocchi’s study [40], neurons were under normal culture condition. Meanwhile, as discussed above, the increased NRSF/REST positive fluorescent particles mainly localized in the nucleus of normal “aging” neurons after relatively long-term culture, whereas elevated in the cytoplasm with abnormal punctates in the HGP-treated senile neurons. Therefore, these results indicated that the transcriptional factor NRSF/REST might regulate cellular senescence with differential patterns between physiological cellular senescence caused by aging and pathological cellular senescence caused by diabetes. By down-regulating the NRSF/REST, senescence-like phenotypes induced by HGP treatment have been successfully alleviated in neurons. This suggests caution in discussing the role of NRSF/REST in cellular senescence. Maintaining the proteostability of NRSF/REST seems to be more beneficial for regulating the senescent cell burden.

Together, in the present study, we used HGP to establish an in vitro diabetic model on neurons and observed obvious senescence-like phenotypes under HGP, which mediated by increased expression of NRSF/REST and impairment of autophagy. Downregulation of NRSF/REST can improve neurosenescence by inhibiting mTOR and promoting autophagy. Our results provide new explanation for impaired proteostasis in the process of diabetic brain aging and raise a new therapeutic target for reducing brain aging caused by diabetes.

## Supplementary Information

**Additional file 1: Figure S1.** CCK-8 assayed neurons viability (a) Detect the viability of PCNs. (b) Detect the viability of PC 12 cells. Data are mean ± SD. n = 5 independent experiments. *p < 0.05, **p < 0.01, ***p < 0.001, ****p < 0.0001. One-way ANOVA for (a) and (b).

**Additional file 2: Figure S2.** Experimental process.

## Data Availability

The original contributions presented in the study are included in the article material, further inquiries can be directed to the corresponding authors.

## Abbreviations

SA-β-gal: Senescence-associated β-galactosidase
NRSF /REST: Neuron restrictive silencer factor / repressor element-1-silencing transcription factor
HGP: High glucose and palmitic acid
PCNs: Primary mouse cortical neurons
γ-H2A.X: Serine139 phosphorylated H2A.X
mTORC: MTOR complex
ULK: Autophagy-initiated UNC-5-like kinase
ATG13: Autophagy related gene 13
