# Supplementary figures and images for: High glucose and palmitic acid induces neuronal senescence by NRSF/REST elevation and the subsequent mTOR-related autophagy suppression
Source: Mol Brain. 2022 Jul 18;15:61. doi: 10.1186/s13041-022-00947-2 (PMC9290252; doi:10.1186/s13041-022-00947-2)

(a)

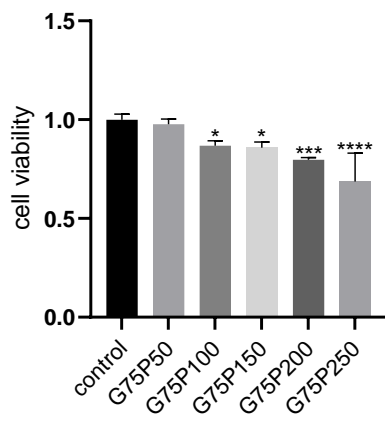

(b)

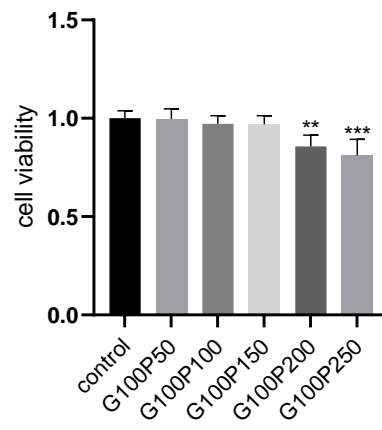

Supplement: Supplementary file 1 — Additional file 1: Figure S1. CCK-8 assayed neurons viability (a) Detect the viability of PCNs. (b) Detect the viability of PC 12 cells. Data are mean ± SD. n = 5 independent experiments. *p < 0.05, **p < 0.01, ***p < 0.001, ****p < 0.0001. One-way ANOVA for (a) and (b). [file 13041_2022_947_MOESM1_ESM.pdf]

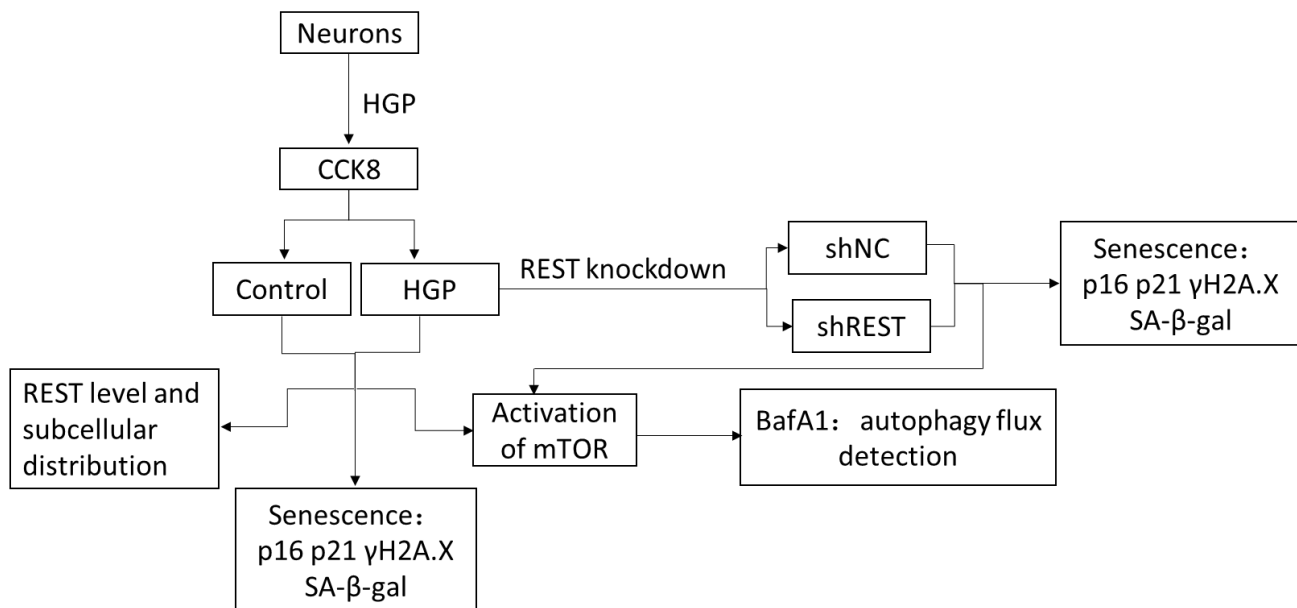

Supplement: Supplementary file 2 — Additional file 2: Figure S2. Experimental process. [file 13041_2022_947_MOESM2_ESM.pdf]
